# Effect of Selenium Supplementation on Redox Status of the Aortic Wall in Young Spontaneously Hypertensive Rats

**DOI:** 10.1155/2015/609053

**Published:** 2015-09-20

**Authors:** Boryana Ruseva, Milena Atanasova, Reni Tsvetkova, Tatyana Betova, Margarita Mollova, Margarita Alexandrova, Pavlina Laleva, Aneliya Dimitrova

**Affiliations:** ^1^Department of Physiology, Medical University, 1 Kliment Ohridski, 5800 Pleven, Bulgaria; ^2^Department of Biology, Medical University, 1 Kliment Ohridski, 5800 Pleven, Bulgaria; ^3^Department of Common and Clinical Pathology, Medical University, 1 Kliment Ohridski, 5800 Pleven, Bulgaria; ^4^Institute of Biology and Immunology of Reproduction, Bulgarian Academy of Sciences, 73 Tsarigradsko Shose, 1113 Sofia, Bulgaria; ^5^Department of Biophysics, Medical University, 1 Kliment Ohridski, 5800 Pleven, Bulgaria; ^6^Central Clinical Laboratory of University Hospital, 8 Georgi Kochev, 5800 Pleven, Bulgaria; ^7^Department of Pathological Physiology, Medical University, 1 Kliment Ohridski, 5800 Pleven, Bulgaria

## Abstract

Selenium (Se) is an exogenous antioxidant that performs its function via the expression of selenoproteins. The aim of this study was to explore the effect of varying Se intake on the redox status of the aortic wall in young spontaneously hypertensive rats (SHR). Sixteen male Wistar Kyoto (WKY) rats and nineteen male SHR, 16-week-old, were tested after being given diets with different Se content for eight weeks. They were divided into 4 groups: control groups of WKY NSe and SHR NSe on an adequate Se diet and groups of WKY HSe and SHR HSe that received Se supplementation. The Se nutritional status was assessed by measuring whole blood glutathione peroxidase-1 (GPx-1) activity. Serum concentration of lipid hydroperoxides and serum level of antibodies against advanced glycation end products (anti-AGEs abs) were determined. Expression of GPx-1 and endothelial nitric oxide synthase (eNOS) were examined in aortic wall. Se supplementation significantly increased GPx-1 activity of whole blood and in the aortas of WKY and SHR. Decreased lipid peroxidation level, eNOS-3 expression in the aortic wall, and serum level of anti-AGEs abs were found in SHR HSe compared with SHR NSe. In conclusion, Se supplementation improved the redox status of the aortic wall in young SHR.

## 1. Introduction

Recent years have witnessed an increased interest in the role of free radical processes in physiology and pathophysiology of the organism. A greater understanding of the mechanisms of production and elimination of reactive oxygen species (ROS) and identification of factors that control or modulate them may help develop relevant strategies for prevention and treatment of ROS-mediated disease states.

Many scientists have been considering the possibility of modulating the progression of hypertension by using antihypertensive drugs and diets within the specific life stages when the organism is most sensitive to endogenous and exogenous factors. Genetically determined hypertension of spontaneously hypertensive rats (SHR) could be used as a model for investigations of the period of life when temporary treatment with adequate drugs or diets may prevent a cardiovascular system from developing pathological changes. Prepubertal and pubertal periods (the age between 4th and 10th weeks of life) are the appropriate periods for the short treatment of SHR by antihypertensive drugs and diets, because at this time intervention is able to have a long-term effect on the development of the cardiovascular system and on the reorganization of hemodynamics [1].

The endothelium is a dynamic structure that has a main importance for maintenance of normal function of cardiovascular system, because of release of vasoactive substances. It is evaluated that abnormal redox state of an arterial wall during atherosclerosis and hypertension in animal models has the most important role in disturbance of vasomotor tone. Endothelium is continuously exposed to blood flow, and it is the primary target of oxidant-induced injury.

Selenium (Se) is an exogenous antioxidant that performs its function via the expression of selenoproteins [2]. Glutathione peroxidases (GPx) and thioredoxin reductases are the main selenoproteins expressed in endothelial cells. They participate in the control of vessel tone, maintaining the balance between the superoxide anions (O_2_^−^) and a nitric oxide (NO), controlling the expression of the cell adhesive molecules, the cell apoptosis, the production of eicosanoids, and the activity of cyclooxygenases and lipoxygenases. Tissue expression of selenoproteins depends on daily Se intake. It has been established that a diet containing 0.1 *μ*g Se/g of food is enough for normal growth and reproduction in mammals [3]. According to the Nutrient Requirements of Laboratory Animals (United States Academy of Science), a diet with Se content 0.15 *μ*g Se/g of food maintains optimal development of rats. It has also been found that a diet containing 0.25 *μ*g Se/g of food given as selenomethionine ensures maximum GPix activity in rat tissues [4].

SHR are the appropriate model for studying essential hypertension in humans, a multifactor disease with an onerous hereditary component, strongly influenced by environmental risk factors.

The aim of this study was to explore the effect of selenium supplementation on the redox status of the aortic wall in young SHR after receiving different Se containing diets in the pubertal period of life.

## 2. Material and Methods

The experiment was performed in accordance with the Animal Welfare Act Regulations and was approved by the University Ethics Committee.

### 2.1. Experimental Animals

Sixteen male Wistar Kyoto (WKY) rats with body mass 207.68 ± 7.01 g and nineteen male 8-week-old SHR (Okamoto-Aoki strain) with body mass 195.89 ± 6.17 g were put on the diets with different Se content for 8 weeks. They were divided into 4 groups: control groups of WKY NSe (*n* = 8) and SHR NSe (*n* = 10) on an adequate Se diet (0.11 *μ*g Se/g of food) and groups of WKY HSe (*n* = 8) and SHR HSe (*n* = 9) that received Se supplementation (0.25 *μ*g Se/g). Se was given as selenomethionine. The rats' systolic blood pressure (SBP) was measured indirectly using a tail cuff, and it was 114 ± 6 mm Hg for WKY and 168 ± 6 mm Hg for SHR at the beginning of the experiment. The rats were placed in the single chambers and had water and food* ad libitum*. The daily intake of food was checked for each rat, calculating the difference between the weight of given food in the morning and weight of its residue after 24 hours.

Blood samples were drawn from the abdominal aorta of 16-week-old rats under pentobarbital sodium anesthesia (40 mg/kg intraperitoneally).

### 2.2. Determination of Serum Selenium Concentration

Determination of serum Se concentration was performed using flameless atomic absorption analysis (Perkin-Elmer, Model Analyst 300).

### 2.3. Assessment of Selenium Nutritional Status

The Se nutritional status was assessed by measuring GPx-1 activity of whole blood at Central Clinical Laboratory of University Hospital, using the “RANSEL” kit according to the protocols of Randox Laboratories Ltd. (Antrim, UK), based on the spectrophotometric method of Paglia and Valentine, and performed by the biochemical analyzer “Hitachi 704” (Japan). The hemoglobin (Hb) content of the blood was determined as well, and GPx-1 activity was given in units: U/g Hb.

### 2.4. Determination of the Serum Concentration of Lipid Hydroperoxides (ROOH)

The serum concentration of lipid hydroperoxides (ROOH) in nmol/mL was evaluated by the spectrofluorometric method of Yagi [5] at the Laboratory of Department of Biophysics.

### 2.5. Indirect ELISA for Determination of the Serum Level of Antibodies against Advanced Glycation End Products (Anti-AGEs Abs)

Keyhole Limpets Hemocyanin (KLH, Sigma-Aldrich, St. Louis, MO, USA) glycated* in vitro *(AGE-KLH) was used as an antigen [6]. Blocking with 0.1% bovine serum albumin (Sigma-Aldrich, St. Louis, MO, USA) was performed. The tested sera diluted 1 : 40 were used as the first antibody. The second antibody was an anti-rat IgG peroxidase conjugate (Sigma-Aldrich, St. Louis, MO, USA) diluted 1 : 2500 with added 1% human serum albumin (Maimex-Bul Bio, Sofia, Bulgaria), incubated for 1 hour at 37°C. The serum samples were assayed at 492 nm on an automatic micro-ELISA plate reader (Ceres UV 900 C, BioTek Instruments Inc., Winooski, VT, USA) at the Immunological Laboratory of Biology Department of Medical University, Pleven.

### 2.6. Histological Investigations

The aortas of the rats, separated into 2 parts (thoracic and abdominal), were extirpated and then fixed in a 10% solution of Formaldehyde and embedded into paraffin blocks. Longitudinal slices (4 *μ*m thick) were prepared and stained by hemalaun-eosin (HE). The morphological changes were then examined and described under light microscope.

Adhesion of slides from aortas was also made for determination of enzyme expression at the Laboratory of Pathomorphology of the Department of Clinical Pathology of Medical University, Pleven. Tissue sections for slides were 5 *μ*m thick for immunofluorescence staining and 3 *μ*m thick for immunohistochemical investigation.

### 2.7. Determination of the Expression of GPx-1 in the Aortic Wall

Immunofluorescence cell staining was performed using kits and protocols of Santa Cruz Biotechnology, Inc. (Santa Cruz, California, USA).

Deparaffinization was performed by xylenes using two changes for 10 minutes each at 40°C water bath. Hydration of sections was performed gradually through graded alcohols (100%, 96%, 85%, and 75%) for 5 minutes each and distilled water. The specimens were incubated with 10% normal blocking serum (normal donkey sera: sc-2044) to suppress nonspecific binding of IgG. The next step was incubation with primary antibody (GPx-1/H-19/goat polyclonal antibody: sc-22146). For a negative control, sections were prepared without the primary antibody. Afterwards, the slides were incubated in a fluorochrome conjugated secondary antibody (donkey anti-goat IgG-FITC: sc-2024). Photos were taken, and the results compared using a semiquantitative analysis of changes under a laser scanning confocal microscope “Leica TCS SPE” (405 nm exiting wale ray; ACS APO 40,0 × 15; immersing objective) of “Leica Microsystems,” Wetzlar, Germany.

### 2.8. Determination of the Expression of Endothelial Nitric Oxide Synthase (eNOS-3) in the Aortic Wall

After the adhesion and deparaffinization of 3 *μ*m thick slides from the aortas, immunohistochemical investigation of the expression of the enzyme eNOS-3 was performed, using kits and protocol from Santa Cruz Biotechnology, Inc. (Santa Cruz, California, USA). The primary rabbit polyclonal IgG antibody NOS3(C-20) and rabbit ABC Staining System were used. Semiquantitative assessment of the degree of expression of eNOS-3 was performed at the immunohistological laboratory under a light microscope, based on the intensity of the stained slides, using the following scale: 0, missing staining, 1, weak staining, 2, moderate staining, and 3, strong staining.

### 2.9. Statistical Methods

The statistical package for social sciences (SPSS) version 15.0 was used for data analysis (SPSS Inc., Chicago, IL, USA). The significance of the differences between groups was assessed by Fisher's* F*-test (ANOVA), multiple range tests (LSD), and *χ* ^2^-test of Pearson at the *P* < 0.05 confidence level. Regression analysis also was made.

## 3. Results

No differences, depending on applied diet, were found for SBP of Wistar rats (SBP = 114 ± 5 and 114 ± 7 mm Hg, resp.) and of SHR (SBP = 168 ± 4 and 170 ± 8 mm Hg, resp.).

**Figure 1:**
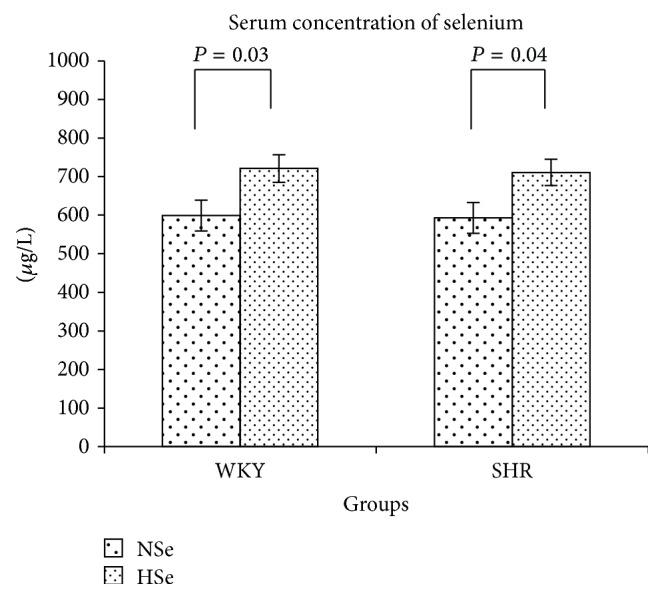
Serum selenium concentration in WKY rats and SHR received varying selenium content diets as the means ± SE (*μ*g/L).

The rats from WKY NSe group had serum Se concentration 599 ± 40 *μ*g/L, those from WKY HSe had 721 ± 35 *μ*g/L, SHR NSe had 592 ± 36, and SHR HSe had 712 ± 34 *μ*g/L. The rats put on a diet supplemented with selenium had significantly higher serum Se concentration (Figure 1).

**Figure 2:**
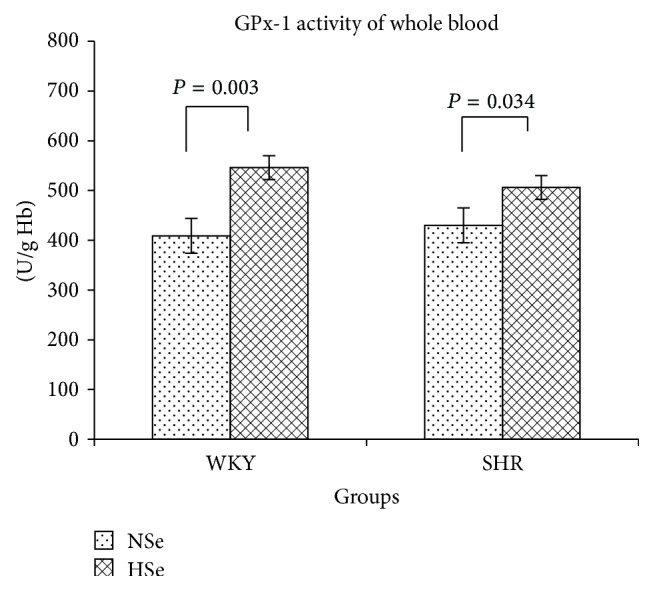
GPx-1 activity of whole blood in WKY rats and SHR received varying selenium content diets as the means ± SE (U/g Hb).

The results showed an increased gpx-1 activity of whole blood in Se supplemented rats as compared to rats receiving an adequate Se diet (Figure 2). The obtained data as means ± SE (U/g Hb) were as follows: for WKY NSe = 409 ± 40, WKY HSe = 546 ± 35 and SHR NSe = 430 ± 24, SHR HSe = 506 ± 24. A moderately strong positive relationship between GPx-1 activity and serum selenium concentration was established.

For WKY rats (*P* = 0.0008),

 GPx-1 = 114.243 + 0.5356  [Se]; *r* = +0.652; *R* ^2^ = 42.46%; SE = 89.54.

For SHR (*P* = 0.0067),

 GPx-1 = 259.824 + 0.3341  [Se]; *r* = +0.615;  *R* ^2^ = 37.77%; SE = 56.19.

Selenium supplementation significantly increased GPx-1 activity of whole blood.

**Figure 3:**
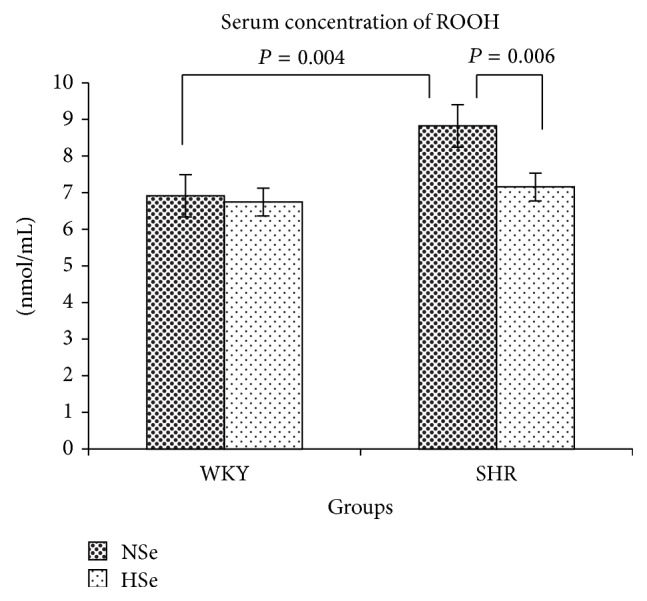
Lipid hydroperoxides serum concentrations of WKY and SHR on varying selenium content diets as the means ± SE (nmol/mL).

The serum concentration of ROOH in rats as means ± SE in nmol/mL was 6.92 ± 0.68 for WKY Nse group and 6.75 ± 0.68 for WKY HSe group and it was 8.83 ± 0.37 for SHR NSe group and 7.16 ± 0.40 nmol/mL for SHR HSe group. SHR NSe had higher serum concentration of ROOH compared to the control WKY group (*P* = 0.004; *F* = 12.46). Selenium supplementation significantly decreased lipid peroxidation level in SHR (*P* = 0.006; *F* = 10.20) but did not affect it in normotensive rats, Figure 3.

Performed regression analysis showed moderate positive relationship (*P* = 0.085) between SBP and serum concentration of ROOH in SHR:

 SBP = 156.52 + 1.84  [ROOH];  *r* = +0.358;  *R* ^2^ = 12.84%;  SE = 7.13.

**Figure 4:**
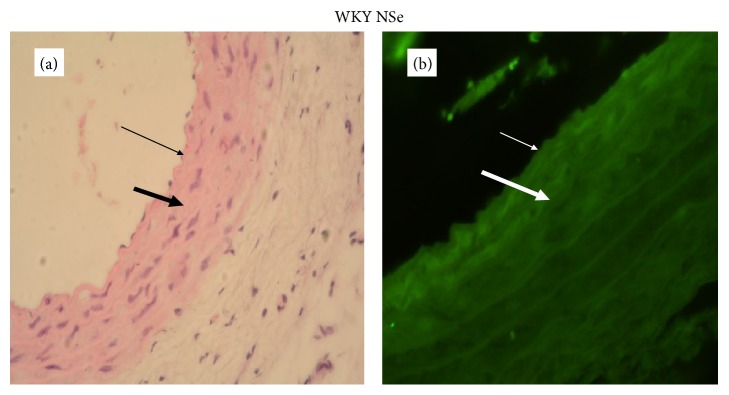
A representative photomicrograph of aortic wall of WKY on adequate selenium content diet (thin arrow: endothelium; thick arrow: media). (a) Histology, intact endothelium and normal thickness of aortic wall (magnification ×400). (b) Expression of GPx-1, immunofluorescent lighting with moderate intensity (magnification ×400).

**Figure 5 fig5:**
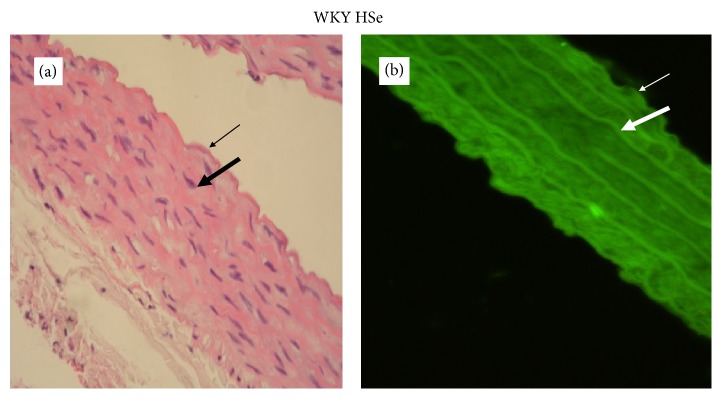
A representative photomicrograph of aortic wall of WKY on high selenium content diet (thin arrow: endothelium; thick arrow: media). (a) Histology, intact endothelium and normal thickness of aortic wall (magnification ×400). (b) Expression of GPx-1, immunofluorescent lighting with high intensity (magnification ×400).

**Figure 6 fig6:**
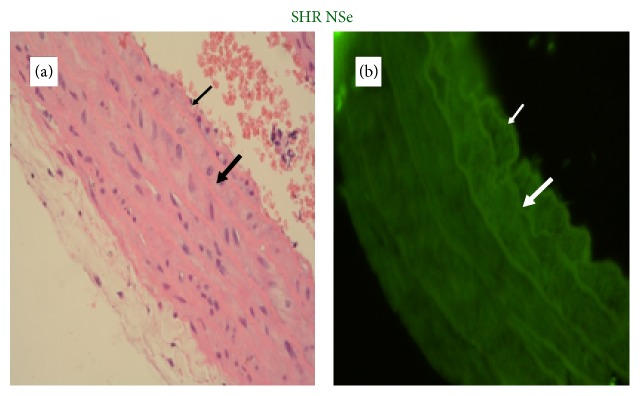
A representative photomicrograph of aortic wall of SHR on adequate selenium content diet (thin arrow: endothelium; thick arrow: media). (a) Histology, roughness of endothelium and normal thickness of aortic wall (magnification ×400). (b) Expression of GPx-1, immunofluorescent lighting with moderate intensity (magnification ×400).

**Figure 7:**
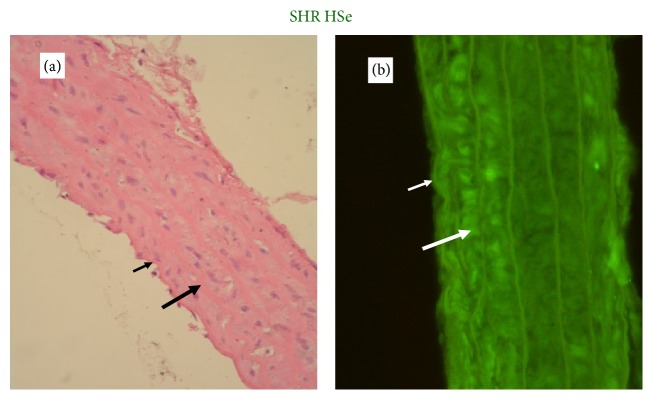
A representative photomicrograph of aortic wall of SHR on high selenium content diet (thin arrow: endothelium; thick arrow: media). (a) Histology, focal loss of endothelial cells and normal thickness of aortic wall (magnification ×400). (b) Expression of GPx-1, immunofluorescent lighting with high intensity (magnification ×400).

The degree of determined GPx-1 expression in the aortic wall depended proportionally on the selenium content of the applied diets (Figures 4 [Fig fig5] [Fig fig6]–7).

**Figure 8:**
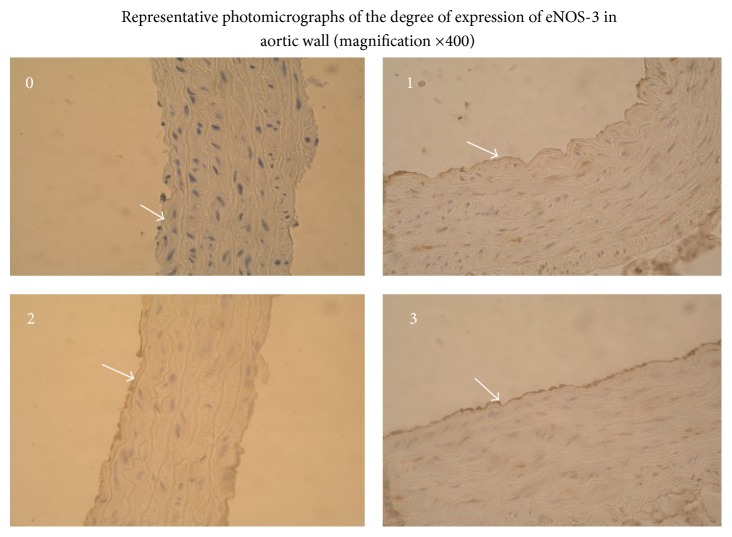
The representative photomicrographs of the degree of eNOS-3 expression in aortic wall of WKY and SHR on varying selenium content diets assessed semiquantitatively using the scale: 0, missed; 1, weak; 2, moderate; 3, high expression (magnification ×400).

The enzyme eNOS-3 in the endothelial cells of the aortic walls was expressed in varying amounts in different slices, from missing to pronounced (Figure 8).

**Figure 9:**
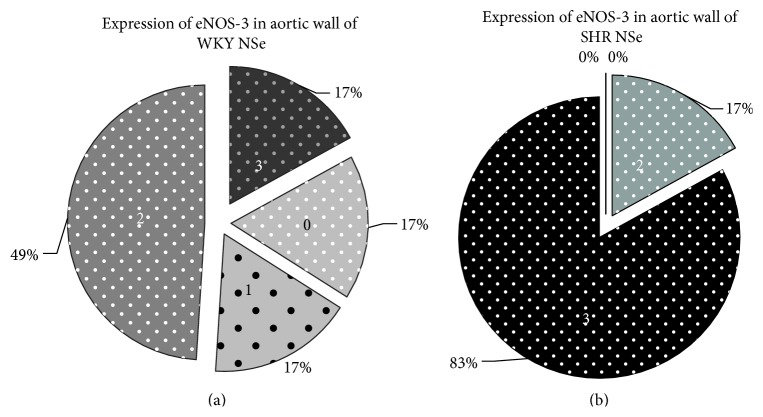
Higher degree of eNOS-3 expression at aortic wall in SHR NSe group compared with WKY NSe group (*P* = 0.035; *χ* ^2^ = 8.57; Pearson's *R* = −0.70). (a) The degrees of eNOS-3 expression in aortic wall of WKY that received adequate selenium content diet were assessed semiquantitatively using the scale: 0, missed; 1, weak; 2, moderate; 3, high expression. (b) The degrees of eNOS-3 expression in aortic wall of SHR that received adequate selenium content diet were assessed semiquantitatively using the same scale.

**Figure 10:**
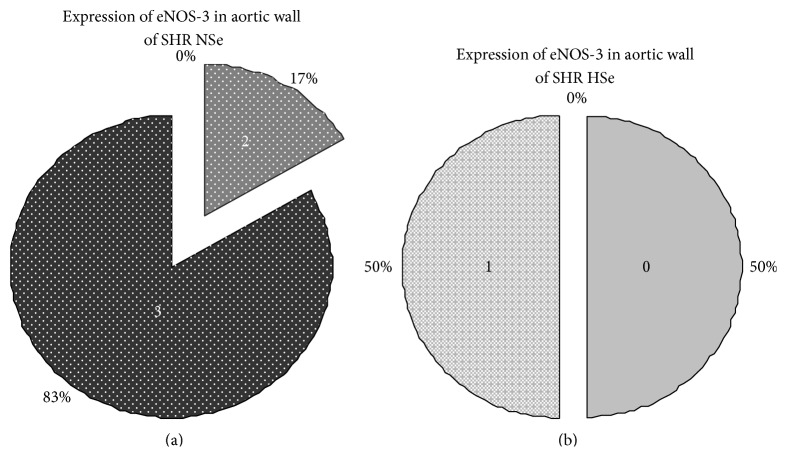
Lower degree of eNOS-3 expression at aortic wall in SHR group that received high selenium content diet compared with SHR group that received adequate selenium content diet (*P* = 0.003; *χ* ^2^ = 18; Pearson's *R* = 0.97). (a) The degrees of eNOS-3 expression in aortic wall of SHR that received adequate selenium content diet were assessed semiquantitatively using the scale: 0, missed; 1, weak; 2, moderate; 3, high expression. (b) The degrees of eNOS-3 expression in aortic wall of SHR that received high selenium content diet were assessed semiquantitatively using the same scale.

We assessed the reported results between groups using Chi-Square test of Pearson and found statistically significant differences: higher degree of expression of eNOS-3 in SHR NSe group compared with WKY NSe group (*P* = 0.035) and lower degree of eNOS-3 expression in SHR HSe group compared with SHR NSe group (*P* = 0.003), Figures 9 and 10.

**Figure 11:**
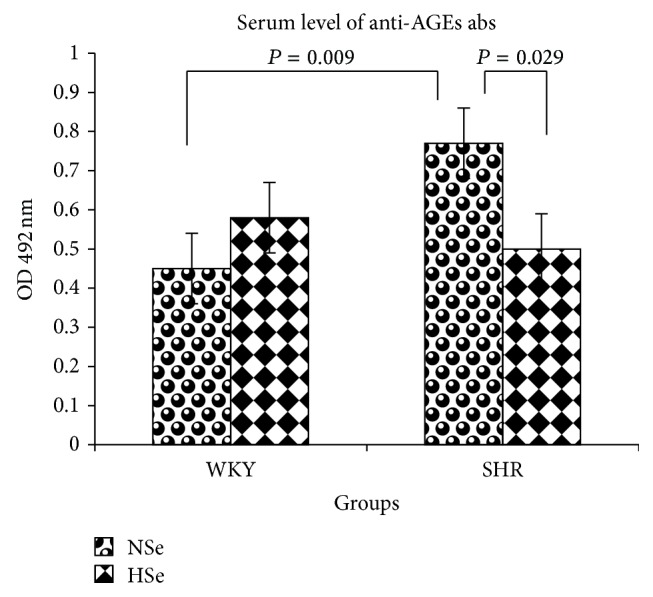
Serum levels of anti-AGEs antibodies in WKY rats and SHR on varying selenium content diets as the means ± SE (OD 492 nm).

Serum concentration of anti-AGEs abs in rats, given as mean ± SE (OD 492 nm), was as follows: 0.45 ± 0.09 in WKY NSe group and 0.58 ± 0.05 in WKY HSe group and 0.77 ± 0.09 in SHR NSe group and 0.50 ± 0.09 in SHR HSe group. The rats from group SHR NSe had higher serum level of anti-AGEs abs compared with WKY NSe (*P* = 0.009; *F* = 8.53). Se supplementation reduced serum level of anti-AGEs abs in SHR (*P* = 0.029; *F* = 5.64) (Figure 11).

## 4. Discussion

The possibility of modulating the progression of diseases with reactive oxygen species-related pathogenesis has been widely discussed in the literature. It has been established that the distribution of Se in the serum of healthy individuals is as follows: 53 ± 6% is bound to selenoprotein P (Sel P), 39 ± 6% is bound to GPx, and 9 ± 4% is albumin-bound [7]. Se supplementation has been suggested to increase the synthesis of selenoproteins because of the increased level of Sec-tRNA, leading to more efficient selenocysteine incorporation. However, the low dietary Se content in humans decreases the serum level of Sel* P* and reduces the GPx-1 activity even more [8].

The scientists measured the GPx-1 activity of the different organs of the rats receiving 0.1 *μ*g Se/g of food and discovered that it was highest in the liver, erythrocytes, lungs, and heart. 98% of the whole blood activity of GPx-1 (528 ± 20 U/g Hb) was due to GPx-1 activity of the erythrocytes, 518 ± 21 U/g Hb [9].

The results obtained in our experiment showed that selenium supplementation significantly increased the GPx-1 activity of whole blood. We also found that only the rats from HSe groups had normal GPx-1 activity in whole blood. The GPx-1 activity of rats from WKY H Se group was 25% greater than that of WKY NSe and in SHR HSe group it was 14% more than that of SHR NSe group. Se supplementation resulting in the increase of the GPx-1 activity of whole blood was also found to decrease the lipid peroxidation serum level in SHR.

Many clinical investigations have shown that the antioxidant enzymes GPx-1 and superoxide dismutase (SOD) have played a central role in the elimination of ROS from the cells. GPx-1 activity of erythrocytes has a prognostic consequence for cardiovascular diseases (CVD), since a significant negative relationship between the risk of heart attacks and determined GPx-1 activity has been shown to exist [10].

GPx-1 is an antioxidant enzyme, catalyzing the reduction of hydrogen peroxide (H_2_O_2_) and the other organic peroxides [11]. It has been reported that a deficit of GPx-1 directly increases oxidative stress levels with consequent endothelial dysfunction [12]. Hydrogen peroxide modulates different aspects of endothelial cell functioning: their growth and proliferation, apoptosis, endothelial-dependant vasodilatation, barrier function, endothelial inflammatory response, and endothelial control on vessel remodeling [13, 14].

The assessment of GPx-1 expression in aortic walls showed that it was directly related to the dietary Se content. Se supplementation increased the fluorescence in the aortic wall. The results showed that GPx-1 was mainly expressed in endothelium and weakly in the media of the aortic wall, but it was not possible to differentiate clearly the level of immunofluorescence between the endothelium and smooth muscle cells in rats with Se supplementation.

From the results obtained, it is evident that the diet containing 0.25 *μ*g Se/g of food significantly increases activity of GPx-1 in the aortic wall as well as in the whole blood of the rats.

Histological examination of the aortic walls showed light pathological changes in rats from group SHR NSe, roughness of endothelium that may disturb hemodynamics in the future.

Excessive production of ROS or inadequate activity of endogenous antioxidant enzymes is the main pathogenetic mechanisms for the development of hypertension-induced damage of target organs. Previously published data showed that low GPx-1 activity in physiological conditions can be compensated for by the intake of vitamin C and vitamin E. However, when the organism is under additional stress factors, use of Se supplementation was safer and more effective in reducing target organ damage [15].

Nitric oxide, synthesized by the enzyme eNOS-3 in the endothelium, modulates the tone of blood vessels and endothelium integrity and inhibits the migration and proliferation of smooth muscle cells. NO, synthesized in cardiomyocytes, is responsible for the normal integrity of the myocardium and mediates the strength of myocardial contraction, which depends on myocardial stretching. The importance of the investigations on eNOS-3 expression has increased, because of its participation in the control of blood pressure variability and myocardial remodeling under conditions of hemodynamic preload [16]. The results obtained in our experiment indicated that eNOS-3 was expressed predominately in endothelial cells and only slightly in the smooth muscle cells of the aorta. We found higher degree of eNOS-3 expression at aortic wall in SHR NSe group compared with WKY NSe group. Se supplementation significantly decreased eNOS-3 expression in SHR.

The enzyme eNOS-3 convertibly binds with different protein family partners, regulating eNOS subcellular localization, catalytic function, and biological activity. A great number of extracellular stimuli dynamically control the eNOS-3 functioning and NO bioavailability in vessel walls by posttranslational modifications. The intimate mechanisms of wrong compartmentalization of eNOS-3 have not yet been discovered, and it may suggest that this enzyme uncoupling is responsible for increased H_2_O_2_ production [17].

Chronically increased blood pressure increases oxidative stress levels by enhancing NAD(P)H-oxidase activity [18]. Overproduction of O_2_^−^ could decrease bioavailable NO and increase production of H_2_O_2_. Under physiological conditions NO concentration is too low to compete effectively with O_2_^−^ for SOD, but under pathological conditions NO functions as a prooxidant. Data has been published showing that peroxides, the NAD(P)H oxidase products, are powerful eNOS activators* in vivo* and are responsible for the increased basal levels of NO and H_2_O_2_ in young SHR [19]. The enhanced level of peroxyl radicals could inactivate NO, thus generating peroxynitrites.

H_2_O_2_ has been established to decrease available L-arginine through stimulation of arginase [20]. Hence, a deficit of GPx-1 activity can lead to increased ROS production and decreased NO availability with no change in the eNOS expression. The reduced levels of bioavailable NO increase eNOS expression by a negative feedback control mechanism.

Our results are in accordance with data published by others, who have reported that the antioxidant drug therapy of SHR decreases eNOS expression in vessels, myocardium, and kidney, despite a compensatory enzyme expression, whereas the treatment has no effect in normotensive rats [21]. Experimental data with cell cultures have shown that the increased ROS level increases eNOS-3 expression [22].

The results obtained proved in practice that eNOS-3 expression and NO bioavailability were influenced in a complex way by the activity of GPx-1 in whole blood and the aortic wall and by the serum lipid peroxidation level.

Glycation of long-lived proteins of the vessel wall accelerated the development of complications, because of their lowered splitting ability, leading to endothelial dysfunction, inflammation, and oxidative stress. The nonenzymatic glycation of tissue proteins was one of the basic methods of posttranslational modifications. These modifications occur slowly under physiological conditions, but they are characteristic futures in the pathogenesis of diabetes arterial hypertonia and atherosclerosis. Disturbed glucose and lipid metabolism stimulate the production of aldehydes that react nonenzymatically with free amino and sulfuric (SH) groups of proteins to form stable conjugates [23]. Excessive accumulation of advanced glycation end products (AGEs) changes protein structure and function and plays a role in immunogenesis. Glycated proteins form the common immunological epitopes that increase the production of antibodies against them (anti-AGE abs). Subendothelial AGEs accelerate the migration and chemotaxis of monocytes. Macrophages have a surface specialized receptor for AGEs (AGE-receptor) which evokes different cellular responses and ROS production [24].

The antioxidant enzymes GPx and glutathione reductase (GR) have SH and amino catalytic groups and participate in the control of oxidative stress. Direct modifications of these groups by aldehydes during AGEs formation, or interaction with AGE-modified proteins, may inhibit the enzyme activity, thus increasing oxidative stress levels.

The accumulation of AGEs in the vessel extracellular matrix (ECM) influences bioavailable NO, an important regulator of vascular tone and smooth muscle cells integrity, by two different mechanisms. Firstly, NO production is decreased by the oxidation of the eNOS cofactor tetrahydrobiopterin, which is an SH-dependent enzyme. Secondly, inactivation of the NO produced may take place due to the interaction between NO-radicals and other free radicals formed by the nonenzymatic glycation reactions.

AGEs stimulate the production of angiotensin II (AII) by increasing the activity of angiotensin converting enzyme. AGEs induce expression of the vasoconstrictor endothelin-1 and alteration of endothelial function toward vasoconstriction [25]. The activation of the AGE-receptor by AGEs also leads to an NAD(P)H oxidase-mediated increase in intracellular ROS levels, extracellular H_2_O_2_ concentration, and VCAM-1 expression in human endothelial cells. In addition to this, AGEs can initiate oxidation of LDL-cholesterol fractions. The interaction of AGEs with the components of the vessel wall increases its permeability, procoagulant activity, and ROS generation resulting in endothelial dysfunction, inflammation, and increased oxidative stress [26, 27].

Intense nonenzymatic glycation rate and/or glycoxidation of ECM proteins in big vessels decrease vascular elasticity. The aorta becomes rigid, causing systolic hypertension and eventually heart insufficiency [28]. AGEs accumulate in atherosclerotic plaques of the aorta and their levels are positively related to arterial wall rigidity in hypertensive patients. Also, the serum concentration of AGEs positively correlates with the intima-media thickness of carotid arteries, inflammation, and risk of rupture [29]. Under hypertonic conditions and in senescence the rate of protein glycation increases accumulates in vascular walls, thus stimulating the production of anti-AGE abs.

As previously discussed, AGEs and ROS modulate blood pro/antioxidant status, leading to changes typical for hypertension and atherosclerosis. It is believed that the use of antioxidants could reduce these changes [21, 30].

The results of this study show that SHR fed a high Se diet had significantly lower serum anti-AGEs abs levels than those of the normal Se diet SHR group, probably because of reduced oxidative stress.

There were not established significant differences between group of WKY rats under Se supplementation and group of WKY rats under adequate Se diet for the concentration of ROOH, serum level of anti-AGEs abs, and eNOS-3 expression, despite significant increase of serum Se concentration and GPx-1 activity in whole blood and in aortic wall.

Many human studies have shown the benefit effects of selenium supplementation on the successful male and female reproduction and on autoimmune, allergic, infectious, neoplastic, and degenerative diseases. The reported data for potential benefit effect of Se supplementation on CVD are conflicting from the different regions of the world, because of the different serum Se concentrations of the patients, depending on the different Se content of the soil. It is recommended to make Se supplementation only in the cases when the selenium concentration is less than 70 *μ*g/L. High Se intake may affect adversely the people with adequate to high Se status, because of increased risk of development of diabetes type 2 or because of close intervals of low adverse effect level of dietary Se (mean LOAEL) that was calculated to be about 1540 ± 653 *μ*g/day and the maximum safe dietary Se (mean NOAEL) 819 ± 126 *μ*g/day [31, 32].

## 5. Conclusions

The selenium-supplemented diet during the pubertal period of SHR improved the redox status of the aortic wall and slowed down the onset of pathological changes.

Future clinical investigations are expected to show the beneficial effects of correct Se supplementation in the early stages of cardiovascular diseases development and to monitor Se nutritional status in humans with cardiovascular risk factors, living in regions of low Se contents in soil, for prevention purposes.

## References

[1] Zicha J., Kuneš J. (1999). Ontogenetic aspects of hypertension development: analysis in the rat. *Physiological Reviews*.

[2] Combs G. F. (2001). Selenium in global food systems. *British Journal of Nutrition*.

[3] WHO (1996). Selenium. *Trace Elements in Human Nutrition and Health*.

[4] National Academy of Sciences (1995). Nutrient requirements of the laboratory rat. *Nutrient Requirements of Laboratory Animals*.

[5] Yagi K. (1987). Lipid peroxides and human diseases. *Chemistry and Physics of Lipids*.

[6] Nakayama H., Taneda S., Kuwajima S. (1989). Production and characterization of antibodies to advanced glycation products on proteins. *Biochemical and Biophysical Research Communications*.

[7] Harrison I., Littlejohn D., Fell G. S. (1996). Distribution of selenium in human blood plasma and serum. *Analyst*.

[8] Driscoll D. M., Copeland P. R. (2003). Mechanism and regulation of selenoprotein synthesis. *Annual Review of Nutrition*.

[9] Whanger P. D., Butler J. A. (1988). Effects of various dietary levels of selenium as selenite or selenomethionine on tissue selenium levels and glutathione peroxidase activity in rats. *Journal of Nutrition*.

[10] Blankenberg S., Rupprecht H. J., Bickel C. (2003). Glutathione peroxidase 1 activity and cardiovascular events in patients with coronary artery disease. *The New England Journal of Medicine*.

[11] Moghadaszadeh B., Beggs A. H. (2006). Selenoproteins and their impact on human health through diverse physiological pathways. *Physiology*.

[12] Forgione M. A., Weiss N., Heydrick S. (2002). Cellular glutathione peroxidase deficiency and endothelial dysfunction. *The American Journal of Physiology—Heart and Circulatory Physiology*.

[13] Cai H. (2005). Hydrogen peroxide regulation of endothelial function: origins, mechanisms, and consequences. *Cardiovascular Research*.

[14] Faraci F. M. (2006). Hydrogen peroxide: watery fuel for change in vascular biology. *Arteriosclerosis, Thrombosis, and Vascular Biology*.

[15] Zhou X., Ji W.-J., Zhu Y. (2007). Enhancement of endogenous defenses against ROS by supra-nutritional level of selenium is more safe and effective than antioxidant supplementation in reducing hypertensive target organ damage. *Medical Hypotheses*.

[16] Balligand J. L., Feron O., Dessy C. (2009). eNOS activation by physical forces: from short-term regulation of contraction to chronic remodeling of cardiovascular tissues. *Physiological Reviews*.

[17] Dudzinski D. M., Michel T. (2007). Life history of eNOS: partners and pathways. *Cardiovascular Research*.

[18] Ungvari Z., Csiszar A., Kaminski P. M., Wolin M. S., Koller A. (2004). Chronic high pressure-induced arterial oxidative stress: involvement of protein kinase C-dependent NAD(P)H oxidase and local renin-angiotensin system. *The American Journal of Pathology*.

[19] Zhou X., Bohlen H. G., Miller S. J., Unthank J. L. (2008). NAD(P)H oxidase-derived peroxide mediates elevated basal and impaired flow-induced NO production in SHR mesenteric arteries *in vivo*. *The American Journal of Physiology—Heart and Circulatory Physiology*.

[20] Thengchaisri N., Hein T. W., Wang W. (2006). Upregulation of arginase by H_2_O_2_ impairs endothelium-dependent nitric oxide-mediated dilation of coronary arterioles. *Arteriosclerosis, Thrombosis, and Vascular Biology*.

[21] Vaziri N. D., Ni Z., Oveisi F., Trnavsky-Hobbs D. L. (2000). Effect of antioxidant therapy on blood pressure and NO synthase expression in hypertensive rats. *Hypertension*.

[22] Zhen J., Lu H., Wang X. Q., Vaziri N. D., Zhou X. J. (2008). Upregulation of endothelial and inducible nitric oxide synthase expression by reactive oxygen species. *The American Journal of Hypertension*.

[23] Vasdev S., Gill V., Singal P. (2007). Role of advanced glycation end products in hypertension and atherosclerosis: therapeutic implications. *Cell Biochemistry and Biophysics*.

[24] Konova E., Andreeva H., Petrova P. (2001). Flowcytometric investigation of AGE-specific receptors and AGE-mediated oxidazing activity of human blood cells. *Clinical Application of Immunological Investigations*.

[25] Potenza M. A., Marasciulo F. L., Chieppa D. M. (2005). Insulin resistance in spontaneously hypertensive rats (SHR) is associated with endothelial dysfunction characterized by imbalance between NO and ET-1 production. *The American Journal of Physiology—Heart and Circulatory Physiology*.

[26] Basta G., Schmidt A. M., de Caterina R. (2004). Advanced glycation end products and vascular inflammation: implications for accelerated atherosclerosis in diabetes. *Cardiovascular Research*.

[27] Esper R. J., Vilariño J. O., Machado R. A., Paragano A. (2008). Endothelial dysfunction in normal and abnormal glucose metabolism. *Advances in Cardiology*.

[28] Vasdev S., Gill V. D., Singal P. K. (2006). Modulation of oxidative stress-induced changes in hypertension and atherosclerosis by antioxidants. *Experimental and Clinical Cardiology*.

[29] Vaziri N. D. (2008). Causal link between oxidative stress, inflammation and hypertension. *Iranian Journal of Kidney Diseases*.

[30] Rodriguez-Iturbe B., Zhan C.-D., Quiroz Y., Sindhu R. K., Vaziri N. D. (2003). Antioxidant-rich diet relieves hypertension and reduces renal immune infiltration in spontaneously hypertensive rats. *Hypertension*.

[31] Rayman M. P. (2012). Selenium and human health. *The Lancet*.

[32] Whanger P., Vendeland S., Park Y.-C., Xia Y. (1996). Metabolism of subtoxic levels of selenium in animals and humans. *Annals of Clinical and Laboratory Science*.

